# Bio-inks for skin regeneration: phase-adaptive design, multifunctional platforms, and 4D bioprinting frontiers

**DOI:** 10.3389/fbioe.2026.1919338

**Published:** 2026-09-23

**Authors:** Gita Bahrami, Aiwei Ma, Ruyi Fan, Chenggang Yi

**Affiliations:** 1 Department of Plastic Surgery, The Second Affiliated Hospital of Zhejiang University College of Medicine, Hangzhou, China; 2 Zhejiang University College of Medicine, Hangzhou, China

**Keywords:** 4D bioprinting, immunomodulation, phase-specific bio-inks, scar modulation, skin wound healing

## Abstract

Chronic wounds and scarring represent major clinical challenges owing to dysregulated tissue repair processes. The emergence of 3D bioprinting offers a promising approach by enabling the fabrication of biomimetic skin substitutes that enhance chronic wound management and facilitate scar modulation. This review provides a phase-oriented framework linking bio-ink properties with the dynamic biological requirements of each wound-healing stage, integrating material engineering, regenerative mechanisms, and translational challenges with a particular focus on establishing phase-specific relationships between bioink properties and wound-healing requirements. By synthesizing recent evidence, we identify a critical gap: phase-specific bio-ink design remains largely underdeveloped and is often only implicitly considered in current analyses of healing phases. Furthermore, we highlight emerging 4D bioprinting strategies and propose that adaptive 4D bio-inks, guided by multi-omics datasets, could facilitate the development of stage-responsive systems capable of adapting therapeutic delivery to the evolving biological requirements of wound repair. Such advancements hold considerable potential to contribute to advancing chronic wound treatment and promote improved scar outcomes.

## 1 Introduction

Chronic wounds and pathological scars remain major challenges in regenerative medicine due to the failure of current therapies to restore functional tissue architecture and precisely regulate complex repair mechanisms. As the largest organ of the human body, skin serves as the primary biological barrier against mechanical injury, pathogen invasion, and water loss, while maintaining essential tissue homeostasis. Its integrity is crucial for maintaining internal homeostasis (Eming et al., 2014). However, injuries penetrating the dermis and subcutaneous tissues (such as trauma or surgical incisions) can trigger complex inflammatory responses and repair processes, leading to pathological scar formation characterized by abnormal fibroblast proliferation and excessive extracellular matrix (ECM) deposition (Dzobo et al., 2019). Additionally, chronic wounds, including diabetic wounds, burn wounds, and infected wounds, exhibit delayed healing or even chronic ulceration due to an imbalance in the local inflammatory microenvironment, insufficient blood supply, and abnormal fibroblast function (Han and Ceilley, 2017). These wounds not only increase treatment complexity but also heighten the risk of secondary infections and sepsis, imposing significant physical, psychological, and financial burdens on patients (Sen, 2019). Chronic wounds impose substantial healthcare costs worldwide, creating a significant economic burden for patients and healthcare systems (Nussbaum et al., 2018). Therefore, effective wound treatment strategies are of paramount importance for promoting skin regeneration and reducing scar formation.

Conventional clinical approaches, however, face significant limitations. In clinical wound management, early wound management strategies relied mainly on protective dressings, but excessive dehydration could disrupt tissue hydration and impair healing. Subsequent incorporation of antimicrobial agents into dressings improves infection control but introduces additional challenges—repeated dressing changes often inflict mechanical trauma on fragile granulation tissue, further delaying the repair process (Junker et al., 2013). The later introduction of moist healing theory, which maintains an appropriate wound microenvironment to promote epidermal cell migration and re-epithelialization, represented a conceptual advance (Wang Y. et al., 2022), with clinical trials further confirming the applicability of the moist healing theory in human tissues (Gibran et al., 2007). Building on these insights, modern biomaterial engineering has developed wound dressings with active regulatory functions that maintain physiological moisture and integrate key features like antibacterial activity and promotion of angiogenesis, significantly improving healing outcomes in complex wounds (Masri and Fauzi, 2021). For scar management, common techniques—including surgical excision, laser ablation, corticosteroid injections, and radiotherapy—largely focus on morphological correction after scar formation. They lack dynamic intervention in early pathological processes such as inflammation imbalance, oxidative stress, and fibrotic signaling, resulting in limited efficacy and high recurrence rates. For instance, corticosteroids can suppress collagen synthesis but may cause skin atrophy, while surgical excision can inadvertently exacerbate fibrosis due to secondary trauma (Kim, 2021; Walsh et al., 2023).

Despite these advances, even the latest wound dressings fall short of addressing the dynamic complexity of pathological scarring and chronic wound repair. They frequently fail to align with the spatiotemporal demands of tissue regeneration, particularly in regulating inflammatory cascades, oxidative stress, angiogenesis, and fibroblast differentiation (Huang et al., 2021). In this context, bio-ink-based 3D bioprinting has emerged as a transformative regenerative strategy. By integrating bioactive components—such as cells, growth factors, and biomimetic matrices—into customizable scaffolds, bioprinting offers unprecedented precision in reconstructing layered skin structures (Pereira et al., 2013). Unlike traditional biomaterials, bio-inks can replicate the extracellular microenvironment and enable localized delivery of immunomodulatory signals, dynamically regulating the healing process (Huang et al., 2021). Moreover, their programmable nature allows for patient-specific designs, such as vascularized dermal–epidermal junctions or gradient-structured matrices, which are critical for accelerating re-epithelialization and restoring function in diabetic or burn wounds (Vijayavenkataraman et al., 2016).

Nevertheless, despite notable progress, current bio-ink strategies remain predominantly material-oriented, focusing on improving printability, mechanical strength, or general biocompatibility. Wound healing, however, is a highly dynamic biological process in which cellular behavior, immune activity, ECM deposition, and mechanical requirements continuously change over time. Therefore, bio-inks designed for skin repair should not only reproduce tissue architecture but also provide stage-dependent biological functions that correspond to the evolving needs of each healing phase—hemostasis, inflammation, proliferation, and remodeling. Establishing a biologically guided framework for selecting and engineering bio-inks according to these phases may represent a critical step toward improving regenerative outcomes and reducing pathological scar formation. In this review, we define phase-specific bio-ink design as the rational matching of bio-ink properties with the dominant biological events, cellular activities, and therapeutic goals of each wound-healing stage, rather than applying a one-size-fits-all material strategy throughout the entire repair process.

While several recent reviews have summarized bio-ink materials, 3D bioprinting technologies, and their applications in skin tissue engineering, most have categorized bio-inks primarily by material composition or fabrication method (Derman et al., 2025; Di Stefano et al., 2025), with comparatively limited emphasis on their stage-specific biological functions throughout the wound-healing cascade. The principal novelty of the present review lies in integrating wound biology with bio-ink engineering through a phase-specific framework that links natural, synthetic, and composite bio-inks to the distinct biological requirements of the inflammatory, proliferative, and remodeling phases. Specifically, this review differs from previous studies by:

1. Establishing a phase-specific framework that connects wound-healing biology with rational bio-ink selection;
2. Critically evaluating how the biological functions of bio-inks, including immunomodulation, angiogenesis, ECM remodeling, and fibrosis regulation, influence outcomes at each stage;
3. Assessing translational barriers that limit clinical implementation, including scalability, manufacturing, regulatory approval, and commercialization challenges.

Pathological scar formation represents a major challenge in achieving functional skin regeneration. In particular, the ability of bio-inks to consistently reduce pathological scarring remains insufficiently validated in human studies. Unlike normal repair, excessive activation of fibroblasts, persistent myofibroblast differentiation, abnormal collagen I/III deposition, and prolonged TGF-β signalling can result in hypertrophic scars and keloids. Therefore, next-generation bio-ink systems should not only accelerate wound closure but also actively regulate fibrosis-related pathways during tissue maturation. Understanding the relationship between wound-healing phases and scar-associated molecular events is essential for designing regenerative biomaterials with improved aesthetic and functional outcomes.

Beyond systematically comparing the advantages and limitations of different bio-ink platforms, we further discuss emerging concepts including phase-specific immunomodulation, adaptive 4D bioprinting, artificial intelligence-assisted biomaterial design, multi-omics-guided bio-ink optimization, and personalized regenerative strategies (Chen et al., 2026). Finally, we critically examine key translational challenges—including large-scale manufacturing, sterilization, regulatory approval, commercialization (Perin et al., 2026), and current clinical progress—to provide a realistic perspective on future clinical implementation. By integrating mechanistic understanding, critical analysis, and translational considerations, this review aims to provide a comprehensive framework for the rational development of next-generation bio-inks capable of promoting functional skin regeneration while minimizing pathological scar formation.

## 2 The molecular mechanisms and phase-specific characteristics of skin wound healing

Wound healing is essential for maintaining skin barrier integrity; disturbances in this dynamic balance can drive chronic wounds or pathological scarring. A clear grasp of the underlying molecular mechanisms and phase-specific features provides the basis for identifying therapeutic targets and designing effective interventions. Healing is a coordinated, multistage process classically partitioned into hemostasis, inflammation, proliferation, and remodelling, each governed by precise temporal and molecular control (Krzyszczyk et al., 2018).

After injury, vasoconstriction limits bleeding and platelets aggregate to form an initial plug; activation of the coagulation cascade converts fibrinogen to fibrin, generating a stable clot that serves as a provisional matrix for subsequent repair (Van De Water et al., 2013). During inflammation, neutrophils clear pathogens and debris and generate ROS; recruited monocytes differentiate into M1 macrophages (e.g., TNF-α, IL-6) and later transition toward a pro-regenerative macrophage phenotype, often characterized by M2-associated markers under anti-inflammatory cues (e.g., IL-4), which promotes angiogenesis and ECM synthesis via factors such as VEGF and TGF-β1, and recruits and activates fibroblasts, thereby bridging to the proliferation phase (Steen et al., 2020). In proliferation, fibroblasts and endothelial cells drive granulation tissue formation, deposit ECM (collagen, fibronectin, hyaluronic acid), and support re-epithelialization; TGF-β1 induces some fibroblasts to become α-SMA^+^ myofibroblasts that contract the wound and restore mechanical integrity (Steen et al., 2020; Mirsky et al., 2024). Remodelling then maintains ECM homeostasis over weeks to months: collagen III is replaced by collagen I, and collagen alignment and cross-linking are regulated by MMPs and TIMPs, contributing to scar maturation; dysregulation (e.g., persistent myofibroblasts or collagen imbalance) results in excessive ECM deposition and hypertrophic scars or keloids (Fernandez-Colino et al., 2019; Balasubramanian et al., 2012). Therefore, controlling fibroblast activation, myofibroblast persistence, and collagen remodelling represents a critical target for scar-modulating bio-ink design.

A concise overview of phase-specific events, cellular players, and mediators is provided in Table 1. Importantly, each wound-healing phase presents distinct biological challenges that require different material functions. During haemostasis and early inflammation, bio-inks should primarily support clot stabilization, antimicrobial protection, and immune regulation. During proliferation, materials should promote cell adhesion, angiogenesis, and ECM deposition, whereas remodelling requires controlled degradation, mechanical support, and regulation of fibrosis. Therefore, understanding phase-specific biological mechanisms provides the rationale for designing and selecting bio-inks with stage-matched regenerative functions.

**TABLE 1:** Key events and cellular players in wound healing phases.

Phase	Key events	Main cells involved	Key molecules	Bio-ink design requirement	Refs
Hemostasis	Clot formation, growth factor release	Platelets, coagulation factors	PDGF, TGF-β, von Willebrand factor	rapid gelation, antibacterial function	(Marston et al., 2019)
Inflammation	Phagocytosis, cytokine release	Neutrophils, macrophages	TNF-α, IL-1, ROS	ROS regulation, immune modulation	(Krzyszczyk et al., 2018; Sharifiaghdam et al., 2022)
Proliferation	ECM deposition, angiogenesis	Fibroblasts, myofibroblasts, epithelial cells	TGF-β, VEGF, collagen	cell adhesion, angiogenesis	(Gibran et al., 2007; Van De Water et al., 2013)
Remodelling	Collagen reorganization, apoptosis	Fibroblasts, myofibroblasts	MMPs, TIMPs, collagen I	mechanical support, fibrosis control	(Steen et al., 2020; Huon et al., 2020)

## 3 Introduction to bio-inks

As a core material in skin tissue engineering, bio-inks provide crucial technical support for constructing artificial skin through their biocompatibility and structural adaptability. Bio-inks are designed to support cell encapsulation, tissue-like architecture fabrication, and regeneration by integrating biological activity with printing performance. Their effectiveness depends on the balance between biocompatibility, printability, and mechanical compatibility with native skin tissue (Mirsky et al., 2024; C Echave et al., 2017). Based on their sources, bio-inks are currently classified into three main categories (Figure 1): naturally derived matrices, synthetic polymers, and composite materials. Natural bio-inks generally provide superior biological signalling and cell compatibility but often suffer from insufficient mechanical stability. Synthetic polymers offer better mechanical control and print fidelity but require functional modification to enhance bioactivity. Composite bio-inks attempt to overcome these limitations by integrating structural stability with regenerative functions.

**FIGURE 1:**
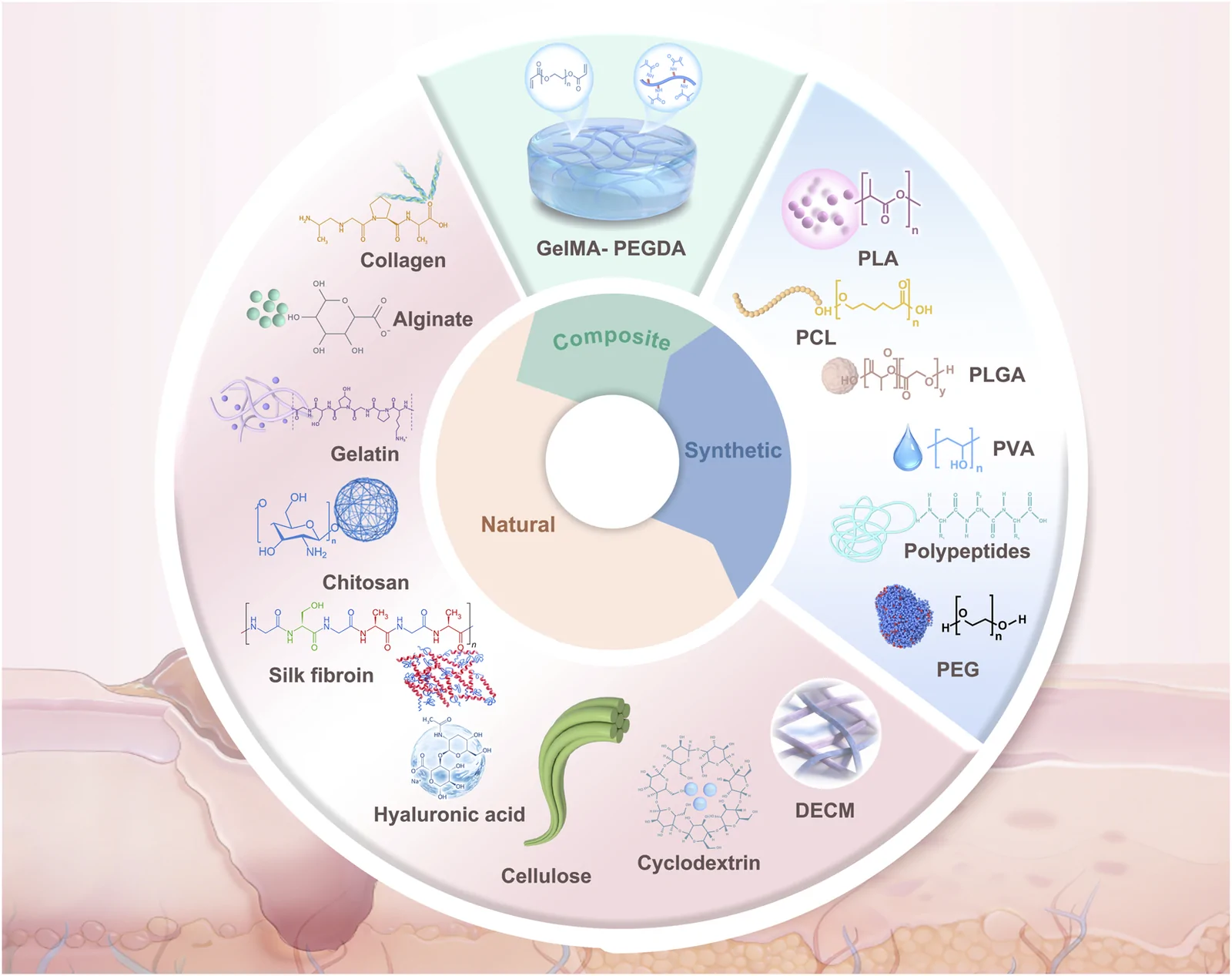
Overview of bio-inks for skin bioprinting, categorized into natural materials (e.g., Collagen, Gelatin, Alginate, Cellulose, Cyclodextrin) and synthetic materials (e.g., PLA, PCL, PLGA, PVA, Polypeptides), used in the fabrication of scaffolds for skin tissue regeneration.

However, material origin alone does not determine clinical suitability, because wound healing proceeds through distinct phases—hemostasis, inflammation, proliferation, and remodeling—each with distinct biological demands. Therefore, bio-ink selection should move beyond conventional classification and instead consider the biological needs, cellular responses, and therapeutic goals of each stage. Table 2 summarises the engineering characteristics, advantages, and limitations of representative bio-inks. Critically, these properties should be evaluated not only as intrinsic material traits but also in terms of their temporal compatibility with the dynamic healing process. For instance, chitosan and alginate are well suited for early phases (hemostasis/inflammation) due to their antibacterial and fluid-management properties, while collagen and synthetic polymers (e.g., PCL, PLGA) provide structural support needed during remodeling. Understanding these trade-offs is essential, as the molecular characteristics and mechanisms of action of each material directly influence wound repair outcomes.

**TABLE 2:** Engineering characteristics, advantages, and limitations governing bio-ink selection for skin tissue engineering.

Bio-ink	Origin/type	Key engineering properties	Main advantages	Major limitations
Collagen	Natural ECM protein	ECM-like structure, biodegradable, cell-interactive	Excellent biocompatibility, supports cell adhesion and matrix formation	Low mechanical strength, rapid degradation without crosslinking
Gelatin/GelMA	Collagen-derived natural polymer	Thermoresponsive behavior, RGD motifs, photocrosslinkable (GelMA)	Good printability, tunable mechanical properties, biological activity	Native gelatin lacks stability; GelMA requires crosslinking optimization
Silk fibroin	Natural fibrous protein	β-sheet structure, high mechanical strength, tunable crosslinking	Excellent toughness, structural stability, suitable for extrusion/DLP printing	Complex processing, limited intrinsic cell signalling
Chitosan	Natural polysaccharide	Cationic polymer, antimicrobial activity, hydrogel formation	Hemostatic activity, antibacterial properties, immunomodulatory potential	Poor solubility, limited elasticity and mechanical strength
Hyaluronic acid	Natural glycosaminoglycan	Highly hydrophilic, ECM-associated, modifiable chemistry	Excellent hydration, cell migration support, ROS regulation	Rapid degradation and weak mechanical stability
Alginate	Natural polysaccharide	Rapid ionic gelation, high water retention	Excellent printability, moisture maintenance, mild gelation conditions	Limited cell adhesion and bioactivity without modification
Cellulose	Natural polysaccharide	Nanofibrillar structure, high mechanical strength	Reinforces scaffolds, improves water retention	Limited biodegradation and biological signalling
Cyclodextrin	Carbohydrate-based material	Inclusion complex formation, molecular encapsulation	Controlled drug delivery and functional modification	Poor structural support alone, requires composite systems
dECM	Decellularized natural matrix	Tissue-specific ECM signals, complex bioactive components	Strong biomimetic properties and regenerative signalling	Batch variability, processing complexity
PLA	Synthetic polyester	High strength, slow degradation, easy processing	Durable scaffolds and structural support	Hydrophobicity and poor cell adhesion
PLGA	Synthetic copolymer	Tunable degradation and drug encapsulation capability	Controlled release and adjustable mechanical properties	Acidic degradation products and limited bioactivity
PCL	Synthetic polyester	High flexibility, slow degradation, excellent printability	Long-term mechanical support and structural stability	Slow degradation and poor hydrophilicity
PVA	Synthetic hydrophilic polymer	Water retention, film formation, hydrogel formation	Maintains moist environment and supports wound coverage	Limited biodegradation and biological activity
PEG	Synthetic hydrophilic polymer	Tunable crosslinking, modular network formation	Precise control of stiffness and degradation	Bioinert nature and poor cell adhesion
Polypeptides	Synthetic biomimetic polymers	Self-assembly, customizable peptide motifs	Programmable biological functions and cell interaction	High cost and manufacturing complexity
Alginate-Gelatin	Natural–natural composite	Ionic/covalent crosslinking, combined bioactivity and printability	Improved mechanics and cell compatibility	Still requires optimization of stability
Collagen-Alginate	Natural–natural composite	ECM mimicry with enhanced mechanical properties	Improved structural integrity and cell support	Limited long-term mechanical reinforcement
GelMA-PEGDA	Natural–synthetic composite	Dual-network formation, tunable mechanics	High stability and controlled therapeutic release	More complex fabrication processes

Therefore, this review proposes that bio-ink selection should not be based solely on material origin (natural vs. synthetic) but should be guided by a phase-specific compatibility principle. This principle posits that the optimal bio-ink for a given wound is the one whose inherent biological and mechanical properties best match the dominant therapeutic requirements of the prevailing healing phase. The following sections will critically evaluate each bio-ink category through this lens, linking their physicochemical characteristics to their potential utility in hemostasis, inflammation, proliferation, or remodeling.

### 3.1 Natural materials

Natural materials mainly originate from biological organisms, including proteins (such as collagen, gelatin, and silk fibroin), polysaccharides (such as chitosan, hyaluronic acid, alginate, cellulose, and cyclodextrin), and decellularized extracellular matrix (dECM). Natural bio-inks often provide excellent biological recognition because they can preserve ECM-derived signals, including cell-binding motifs and biochemical cues that support cellular adhesion, proliferation, differentiation, and tissue remodeling. These characteristics make natural materials highly attractive for skin regeneration, where biological integration is essential for successful repair. However, their clinical translation is frequently limited by insufficient mechanical stability, poor shape fidelity during printing, rapid degradation, and batch-to-batch variability. Therefore, current research increasingly focuses on hybridization and functional modification strategies that combine the biological activity of natural materials with improved printability, structural durability, and spatiotemporal control over therapeutic functions.

#### 3.1.1 Protein-based materials

##### 3.1.1.1 Collagen

Beyond its structural role in native skin, collagen has attracted considerable attention as a bio-ink component due to its ECM-mimicking characteristics, including mechanical support, regulation of cellular interfaces, and transduction of growth factor signals, which play a pivotal role in tissue development and injury repair (Fernandez-Colino et al., 2019). Type I collagen mainly contributes to mechanical stability, whereas type III collagen is associated with early matrix formation and tissue remodeling. However, collagen-based bio-inks used alone often exhibit poor mechanical strength, rapid degradation, and limited shape fidelity during printing, restricting their application in large and complex defects (Shoulders and Raines, 2009; Chattopadhyay and Raines, 2014). Excessive collagen I deposition and altered collagen I/III ratios are associated with pathological scar formation. Recent collagen-based bioinks have demonstrated improved wound healing outcomes by supporting fibroblast activity, ECM deposition, re-epithelialization, and vascularized tissue regeneration in preclinical models (Chen H. et al., 2023). Consequently, current strategies increasingly employ collagen-based composite systems to preserve biological functionality while improving mechanical performance and manufacturing reliability.

Accordingly, collagen is primarily suited for the proliferative and early remodeling phases, where ECM-mimetic signaling and matrix deposition are dominant. However, its clinical application remains dependent on improving mechanical stability through composite strategies.

##### 3.1.1.2 Gelatin and gelatin-methacryloyl (GelMA)

Gelatin, a hydrolysed derivative of collagen, is a naturally occurring polypeptide widely utilized in biomedical applications due to its biocompatibility, biodegradability, and thermoresponsive properties. Gelatin retains collagen-derived bioactive motifs, particularly RGD sequences, which support cell adhesion and regenerative responses. However, native gelatin exhibits limited mechanical stability and rapid dissolution under physiological conditions, which restricts its direct application in structurally demanding wounds (Bello et al., 2020). To overcome these limitations, gelatin has been chemically modified into gelatin methacryloyl (GelMA), a photocrosslinkable derivative that provides improved mechanical tunability while retaining gelatin-derived biological cues. Recent advances in gelatin-based bio-ink formulations aim to address challenges in chronic wound management through improved matrix engineering. For instance, a study developed GelMA bio-ink crosslinked with UV light, which enhanced moisture retention, improved mechanical stability, and preserved ECM-mimicking bioactivity, thereby supporting fibroblast adhesion and ECM synthesis (Yin et al., 2023). Another investigation developed a composite gelatin–alginate bio-ink using an ionic and covalent double-crosslinking strategy, which improved printability, mechanical stability, and cell viability, with *in vivo* studies demonstrating reduced inflammation, enhanced collagen deposition, and improved angiogenesis (Hao et al., 2023). Furthermore, a recent study developed a 3D-printable double-network bio-ink composed of hydrazone-crosslinked hyaluronic acid and photocrosslinked GelMA, demonstrating improved mechanical strength, shear-thinning behaviour, self-healing capability, and support for stem cell viability (Abalymov et al., 2022). Collectively, these advances highlight the transition of gelatin-based bio-inks from passive structural supports toward bioactive and tunable regenerative platforms. Future developments will likely focus on optimizing GelMA-based composite systems to achieve improved mechanical performance, vascularization, and phase-specific regulation of wound healing.

GelMA-based systems are particularly suitable for the proliferative phase, where cell infiltration, angiogenesis, and matrix formation are dominant processes.

##### 3.1.1.3 Silk fibroin

Silk fibroin (SF) is a mechanically robust natural protein with excellent structural stability and printability; however, compared with ECM-derived materials such as collagen and gelatin, its limited intrinsic biological signalling requires functional modification or combination with bioactive components to enhance regenerative functions. SF can be processed using extrusion and light-based printing approaches, with light-assisted methods generally providing improved control over microarchitecture and shape fidelity (Fang et al., 2024). To enable photopolymerization, SF is frequently methacrylated (Sil-MA/SFMA) and combined with a cytocompatible photoinitiator (e.g., LAP) (Kim et al., 2021). Recent studies have developed double-crosslinking strategies for SF-based bio-inks, combining photochemical and enzymatic crosslinking approaches to improve structural stability, degradation control, and cellular compatibility in printed constructs (Agostinacchio et al., 2024). Mechanical tunability can be adjusted through formulation strategies, such as incorporation of methacrylated hyaluronic acid, which improves network stiffness, gelation kinetics, and post-print stability while maintaining cytocompatibility (Amirian et al., 2024). Light-assisted fabrication approaches, including DLP printing, have further expanded SF-based bio-inks by enabling precise microarchitecture control and improved epidermal–dermal patterning (Lai et al., 2024). Overall, SF-based bio-inks represent a mechanically robust but biologically tunable platform for cutaneous repair.

SF-based bio-inks are particularly valuable during the proliferative and remodelling phases, where matrix stabilization and long-term structural support are required.

#### 3.1.2 Polysaccharide and monosaccharides-based materials

##### 3.1.2.1 Chitosan

Chitosan, a linear polysaccharide derived from the partial deacetylation of chitin, is a prominent biomaterial due to its biocompatibility, biodegradability, and inherent antimicrobial properties (Bakshi et al., 2020). Its cationic structure enables interactions with negatively charged cell membranes and microbial surfaces, conferring antibacterial and hemostatic effects critical for wound management (Kou et al., 2021; Lazaridou et al., 2022). Additionally, chitosan’s ability to degrade into non-toxic oligosaccharides and stimulate immune responses—such as macrophage activation and cytokine release—makes it a versatile scaffold material for skin regeneration, particularly in infection-prone environments (Tiburcio et al., 2021). Chitosan–hyaluronic acid composite hydrogels have demonstrated considerable potential in infected wound healing. For example, a study developed a gallic acid–grafted quaternized chitosan/oxidized hyaluronic acid (GA-QCS/OHA) hydrogel that demonstrated strong antibacterial activity, reduced inflammatory responses, enhanced vascularization, and promoted wound closure in infected wound models (Elshabrawy et al., 2025). Recent studies have developed PVA–chitosan composite wound dressings loaded with PHMB, demonstrating sustained antiseptic release and effective antibacterial activity against *Staphylococcus aureus* and S. epidermidis (Taneja et al., 2022; Worsley et al., 2019).

Chitosan-based bio-inks are particularly suitable for haemostasis and early inflammatory phases, where rapid infection control and regulation of early immune responses are critical determinants of successful tissue repair.

##### 3.1.2.2 Hyaluronic acid

Hyaluronic acid (HA), a naturally occurring glycosaminoglycan, is a key component of the ECM in connective tissues, particularly abundant in skin (Xu et al., 2020). HA exhibits excellent water retention, cell-interactive properties, and immunomodulatory potential, making it suitable for supporting inflammation resolution and proliferative regeneration (Chen H. et al., 2023). HA interacts with cell surface receptors such as RHAMM and CD44, modulating cellular processes like migration, proliferation, and inflammation resolution (Litwiniuk et al., 2016). Furthermore, HA’s biodegradability by hyaluronidases, low immunogenicity, and ability to scavenge reactive oxygen species (ROS) position it as an ideal biomaterial for bio-ink development, particularly in applications requiring a moist microenvironment and antioxidant protection (Zhu et al., 2020). A recent study developed a 3D-printable double-network bio-ink of hydrazone-crosslinked HA and photocrosslinked GelMA, demonstrating improved mechanical strength, shear-thinning behaviour, self-healing capability, and suitability for tissue engineering applications (Abalymov et al., 2022). Another investigation developed an HA-based bio-ink crosslinked via methacrylation (HAMA) and blended with collagen, encapsulating adipose-derived stem cells (ASCs), which accelerated wound closure and enhanced vascular regeneration in full-thickness mouse wound models (Mirsky et al., 2024).

HA-based bio-inks are particularly relevant to the proliferative phase, where regulation of inflammation resolution, fibroblast migration, angiogenesis, and ECM deposition are essential. However, similar to many polysaccharide-based systems, HA-based bio-inks often require chemical modification or combination with reinforcing polymers to improve mechanical stability and long-term structural integrity.

##### 3.1.2.3 Alginate

Alginate, a naturally derived polysaccharide extracted from brown seaweed, is widely utilized due to its biocompatibility, rapid gelation properties, and structural similarity to the ECM (Lee and Mooney, 2012). Alginate forms rapidly crosslinked hydrogels with excellent water retention and printability, although its limited cell adhesion requires combination with bioactive materials. Its high water content (up to 95%) and ability to maintain a moist wound environment make it an excellent candidate for bio-ink formulations (Abasalizadeh et al., 2020). Furthermore, alginate’s mild antimicrobial effects in modified formulations and gentle ion-exchange-based gelation make it a versatile bio-ink component for managing highly exudative wounds (Eichhorn, 2011). For example, a study developed an alginate-gelatin bio-ink loaded with VEGF, demonstrating enhanced angiogenesis and wound closure in porcine burn models within 21 days. Another investigation utilized a 3D-printed alginate-chitosan scaffold, which reduced wound area and enhanced collagen deposition in rat wound models. Additionally, an alginate-based bio-ink crosslinked with strontium ions improved fibroblast activity and accelerated re-epithelialization in mouse chronic wound models (Bahú et al., 2022).

Due to its rapid gelation and fluid absorption capacity, alginate-based bio-inks are particularly advantageous during the early stages of wound repair, including haemostasis and inflammatory regulation. However, their limited intrinsic cell-adhesive properties require combination with ECM-derived materials or bioactive molecules to achieve advanced regenerative outcomes.

##### 3.1.2.4 Cellulose

Cellulose, a linear polysaccharide composed of β-(1→4)-linked D-glucose units, is the most abundant natural polymer on Earth, sourced from plant cell walls, cotton, and certain bacteria (Eichhorn, 2011). Its highly crystalline structure imparts exceptional mechanical strength and stability, while its hydrophilic nature supports a moist environment conducive to wound healing (Afewerki et al., 2019; Zheng et al., 2022). Bacterial cellulose (BC), in particular, stands out due to its nanofibrillar network, high purity, and biocompatibility, enabling it to mimic the ECM’s topography and facilitate cell adhesion and proliferation. Additionally, cellulose’s biodegradability, low cytotoxicity, and ability to be functionalized with bioactive molecules enhance its utility in bio-ink formulations (Tiburcio et al., 2021). For example, a cellulose nanofiber-gelatin composite bio-ink enhanced angiogenesis and wound closure in porcine burn models by sustaining a moist microenvironment and supporting vascular growth (Afewerki et al., 2019). Furthermore, electrospun cellulose acetate nanofibres embedded with CuO and silver nanoparticles demonstrated strong broad-spectrum antibacterial activity *in vitro* and favourable cell compatibility (Armstrong et al., 2017).

Cellulose-based bio-inks are mainly valued for their structural reinforcement, moisture regulation, and long-term scaffold stability, making them more suitable for supporting tissue maturation and remodelling rather than directly regulating early cellular responses.

##### 3.1.2.5 Cyclodextrin (CD)

CD, a family of cyclic oligosaccharides derived from starch, has a unique molecular architecture with a hydrophilic exterior and a hydrophobic internal cavity, enabling it to form inclusion complexes with drugs, growth factors, and antimicrobial agents, thereby enhancing their solubility, stability, and bioavailability (Zhang and Ma, 2013). Unlike conventional structural bio-inks, CD-based systems provide a unique advantage as bioactive delivery platforms, allowing temporally controlled release of therapeutic molecules according to the changing requirements of wound healing phases. This host-guest interaction, combined with CD’s biocompatibility, biodegradability, and low toxicity, makes it a promising candidate for bio-ink formulations (Zhang and Ma, 2013). Furthermore, CD’s ability to be chemically modified allows for tailored mechanical properties and degradation profiles. For example, a β-cyclodextrin-hyaluronic acid bio-ink loaded with curcumin demonstrated reduced inflammatory responses and improved wound closure in rat models through sustained drug release. Another investigation utilized a 3D-printed α-cyclodextrin-gelatin composite bio-ink incorporating VEGF, which enhanced angiogenesis and wound closure in porcine burn models (Xue et al., 2023). CD itself provides limited structural support, and its application as a bio-ink generally depends on integration with mechanically stronger hydrogel networks.

Collectively, polysaccharide-based bio-inks provide diverse functional advantages in skin regeneration. Chitosan and alginate are particularly valuable for infection control, haemostasis, and inflammatory regulation due to their antimicrobial and fluid-management properties, whereas HA offers superior biological signalling for cell migration and angiogenesis. Cellulose contributes mechanical reinforcement and structural stability, while cyclodextrin provides advanced therapeutic delivery capabilities. However, most polysaccharide-based bio-inks still require modification or combination with proteins, synthetic polymers, or bioactive factors to overcome limitations in mechanical strength, cell adhesion, and long-term tissue integration.

#### 3.1.3 Decellularized extracellular matrix (dECM)

Decellularized extracellular matrix (dECM) bio-ink is generated by removing cellular components from donor tissues/organs via physical, chemical, and enzymatic treatments while preserving the native ECM architecture and key bioactive cues (e.g., collagens, glycosaminoglycans, growth factors). It offers pronounced tissue specificity and biocompatibility, recreating a near-physiologic microenvironment that directs cell adhesion, migration, differentiation, and tissue regeneration. In skin applications, accumulating evidence indicates that dECM can accelerate re-epithelialization and angiogenesis while modulating the immune milieu (e.g., biasing macrophages toward an M2, pro-healing phenotype) and attenuating scar formation (Xiao et al., 2023; Jiang et al., 2023).

Despite these advantages, dECM still faces practical bottlenecks—time-consuming preparation, batch variability, limited mechanical robustness, and narrow printability windows—necessitating combination with photocrosslinkable support networks to achieve controllable rheology, shape fidelity, and structural stability. For example, coupling dECM with GelMA/HAMA enables rapid light-triggered gelation and improved interlayer adhesion while maintaining pro-regenerative bioactivity (Lai et al., 2024). In translational skin settings, 3D-printed composite bio-inks loaded with human adipose-derived stem cells have demonstrated faster closure, enhanced vascularization, and stratified epidermal-dermal reconstruction in preclinical models (Fu et al., 2023). Overall, a “dECM + photocrosslinkable composite” strategy—using tuned composition ratios and crosslinking kinetics—has emerged as a widely investigated strategy to align bio-ink performance with the spatiotemporal demands of cutaneous repair.

#### 3.1.4 Comparative perspective of natural bio-inks

Natural bio-inks provide superior biological compatibility but differ considerably in their functional roles and mechanical performance. A cross-category comparison reveals consistent trade-offs: protein-based natural bio-inks (collagen, gelatin, GelMA) excel in ECM-mimetic signaling and cell interaction but lack mechanical stability, making them more effective as bioactive components within composite systems rather than as standalone printing materials. Polysaccharide-based natural bio-inks (chitosan, alginate, HA) provide superior hydration, antimicrobial activity, ROS regulation, or fluid management, but generally require reinforcement strategies to overcome limited mechanical stability and biological signalling. Silk fibroin offers superior mechanical stability compared with collagen and gelatin but provides fewer intrinsic biological signals, making composite strategies essential for balancing structural and regenerative functions. dECM-based systems provide the highest level of tissue-specific signalling but face challenges related to standardization and mechanical stability. Therefore, natural bio-inks are increasingly developed as composite systems to balance biological activity, printability, and mechanical performance. However, no single natural bio-ink currently provides optimal biological activity, mechanical stability, printability, and phase-specific functionality simultaneously, supporting the increasing use of composite natural bio-ink systems.

### 3.2 Synthetic materials

Synthetic materials offer distinct advantages in bio-ink development, including precise control over mechanical properties, degradation rates, and functionalization, making them complementary to natural biomaterials in skin tissue engineering (Murphy and Atala, 2014). Unlike natural polymers, synthetic materials can be tailored to meet specific clinical needs, such as enhanced durability or targeted drug release, while avoiding immunogenicity risks associated with animal-derived sources. Their versatility and reproducibility have driven their integration into 3D bioprinting, enabling the fabrication of scaffolds with optimized architectures for wound repair and regeneration (Farah et al., 2016). However, most synthetic polymers are inherently bioinert, lacking cell recognition motifs and thus exhibiting poor cell adhesion. Furthermore, their degradation byproducts may induce inflammatory responses or cytotoxicity, resulting in overall biocompatibility that is typically inferior to that of natural materials. Consequently, surface modification—such as RGD peptide grafting—or combination with natural polymers is commonly employed to enhance their biological activity.

Unlike natural bio-inks that provide intrinsic biological signals, synthetic polymers are primarily valued for their programmable mechanical properties, degradation profiles, and manufacturing reproducibility. Hydrophobic synthetic polyesters (PLA, PLGA, PCL) offer superior mechanical durability and processing stability but lack intrinsic cell-recognition signals, requiring blending with bioactive polymers or surface modification strategies. Hydrophilic synthetics (PVA, PEG) provide excellent moisture retention, tunable crosslinking, and hydration management, but generally require reinforcement strategies or bioactive modification to achieve long-term mechanical stability and effective cell–material interactions. Polypeptides offer programmable biochemical signalling and self-assembly properties, making them highly tunable platforms for integrating biochemical signalling with material properties, though high cost and manufacturing complexity remain barriers. Their application in skin regeneration is determined by the specific requirements of each healing stage: PVA-based systems are advantageous for maintaining hydration during early wound repair; PLGA and PLA provide sustained structural support during tissue maturation; and PEG-based and polypeptide systems offer tunable platforms for controlled delivery and dynamic regulation of cellular responses. However, because synthetic materials generally lack native ECM-derived signalling, successful skin regeneration increasingly relies on hybridization with natural polymers or incorporation of bioactive motifs.

#### 3.2.1 Polylactic acid (PLA)

PLA, a biodegradable aliphatic polyester derived from renewable resources, is widely employed due to its biocompatibility, mechanical strength, and controllable degradation profile (Farah et al., 2016). Its mechanical properties make it suitable for structural support in skin constructs; however, its hydrophobicity and limited cell-recognition capacity often require blending with bioactive polymers or surface modification strategies (Vijayaraghavan et al., 2025). For example, electrospun PLA–gelatin nanofibers conjugated with epidermal growth factor (EGF) demonstrated enhanced fibroblast viability and accelerated wound closure in rat models (Taghizadeh et al., 2022). Additionally, PLA–chitosan electrospun nanofiber dressings incorporating cod liver oil achieved accelerated wound closure in diabetic rat models with improved collagen deposition and angiogenesis (Elshabrawy et al., 2025). PLA is primarily valuable during the remodelling stage, where long-term mechanical support is required, although bioactive modifications are necessary to support earlier regenerative events.

#### 3.2.2 Poly (lactic-co-glycolic acid) (PLGA)

PLGA, a synthetic copolymer of lactic acid and glycolic acid, is widely investigated due to its tunable biodegradability, biocompatibility, and versatility in scaffold design. Synthesized via ring-opening copolymerization, PLGA’s degradation kinetics can be modulated by adjusting the lactide-to-glycolide ratio and molecular weight, with 50:50 formulations generally exhibiting faster degradation (Wu et al., 2021; Omidian and Wilson, 2025). Its amorphous structure provides flexibility and a glass transition temperature ranging from 40° to 60 °C, making it suitable for 3D bioprinting, while its ability to encapsulate hydrophobic drugs and bioactive molecules enhances its utility in controlled release applications. For example, co-axial PLGA–gelatin fibres loaded with SDF-1α and angiogenic peptides enhanced endothelial cell migration, promoted angiogenesis, and improved wound closure in diabetic wound models (Huon et al., 2020). In a porcine partial-thickness burn model, scaffolds incorporating 5% RGD or 10% hyaluronic acid achieved near-complete re-epithelialization by day 21 with markedly reduced inflammation (lin et al., 2024; Teo et al., 2023). Although PLGA’s hydrophobicity may limit initial cell adhesion, functionalization with natural polymers or surface modifications can overcome this. Acidic degradation products generated during PLGA degradation may influence local inflammatory responses, requiring careful optimization for chronic wound applications.

PLGA-based bio-inks may be particularly advantageous during the proliferative and early remodelling phases, where sustained factor delivery and matrix support are required.

#### 3.2.3 Polycaprolactone (PCL)

PCL is a semi-crystalline aliphatic polyester obtained via ring-opening polymerization of ε-caprolactone. Due to its favorable biocompatibility, prolonged degradation behavior, and strong mechanical properties, it has become a widely used synthetic biomaterial in tissue engineering (Woodruff and Hutmacher, 2010). PCL offers flexibility and ease of processing, making it highly suitable for 3D bioprinting applications (Malikmammadov et al., 2018). Its degradation occurs via hydrolysis of ester bonds over an extended period (typically 1–2 years), providing prolonged structural support, though its hydrophobicity can hinder cell attachment unless modified with hydrophilic polymers or bioactive agents (Mokhena et al., 2024). PCL’s high tensile strength (up to 20–40 MPa) enhances its utility in skin regeneration, particularly for applications requiring durable scaffolds (Pugliese et al., 2021). For example, electrospun PCL–gelatin scaffolds treated with fibroblast-conditioned medium enhanced fibroblast adhesion, proliferation, and ECM-related gene expression *in vitro* (Abdollahiyan et al., 2020). Similarly, electrospun PCL–hyaluronic acid scaffolds exhibited a biomimetic nanofibrous architecture that promoted cellular interactions, with enhanced fibroblast adhesion and proliferation compared with pure PCL scaffolds (Radmanesh et al., 2022).

PCL-based systems are particularly relevant to the remodeling phase, where long-term structural support and controlled matrix maturation are required. Consequently, PCL is frequently combined with gelatin, collagen, or HA to balance mechanical reinforcement with biological activity.

#### 3.2.4 Polyvinyl alcohol (PVA)

PVA, a synthetic hydrophilic polymer derived from the hydrolysis of polyvinyl acetate, is extensively utilized due to its excellent biocompatibility, water solubility, and film-forming capabilities (Teodorescu et al., 2018). PVA’s degree of hydrolysis governs its mechanical strength and gelation properties, enabling it to form hydrogels through physical or chemical crosslinking (Teodorescu et al., 2018). Its excellent moisture retention, flexibility, and biocompatibility make it an effective component in wound dressings designed to maintain a moist environment (Taneja et al., 2022). For example, a 3D-printed PVA-gelatin bio-ink achieved enhanced keratinocyte migration and reduced wound area in rat models within 14 days. Another study utilized a PVA–chitosan composite bio-ink loaded with antimicrobial peptides, which reduced bacterial burden and enhanced collagen deposition in infected mouse wound models (Sun et al., 2021).

These hydration-retaining characteristics make PVA-based bio-inks particularly beneficial during early wound healing phases, where maintaining moisture balance and supporting epithelial migration are critical. However, similar to many synthetic hydrophilic polymers, PVA lacks intrinsic cell-recognition motifs and often requires incorporation of bioactive components to enhance tissue integration.

#### 3.2.5 PEG

PEG is a hydrophilic, non-ionic synthetic polymer widely used as a hydrogel matrix because of its protein-repellent surface, aqueous swellability, and highly tunable crosslinking chemistry. In wound applications, PEG is most often deployed as PEG diacrylate (PEGDA) or multi-arm PEG, allowing rapid gelation under mild conditions and precise control over stiffness, mesh size, and degradation. On its own PEG is bioinert, so skin-facing formulations typically integrate bioactive motifs (RGD), natural polymers (e.g., gelatin, collagen, hyaluronic acid), or antimicrobial/anti-inflammatory cargos to enhance cell adhesion, angiogenesis, and infection control while maintaining a moist, conformal barrier. Recent work with injectable alginate/PEG hybrid hydrogels formed via thiol–Michael addition has demonstrated promising performance, including rapid *in situ* setting, hemostatic activity, and accelerated wound closure in animal models (Xie et al., 2025). Beyond injectables, PEG-containing networks feature prominently in advanced hemostatic and wound-dressing designs (e.g., catechol- or choline-modified PEGs, multi-arm PEG sealants), where the polymer’s chemistry supports adhesion to wet tissue, burst-resistance, and controllable proteolytic stability (Zhang et al., 2024). For 3D printing, PEG-based (photo)crosslinkable systems (PEGDA, PEG-thiol-ene) are compatible with extrusion and DLP, enabling thin, conformable lattices or patterned dressings that can spatially organize keratinocytes/fibroblasts and deliver bioactive factors in a spatiotemporally programmed manner (Ribeiro et al., 2024).

However, the intrinsic bioinert nature of PEG remains a major limitation, requiring incorporation of biological motifs or hybrid networks to achieve effective cell–material interactions.

#### 3.2.6 Polypeptides

Polypeptides, synthetic polymers composed of amino acid sequences, are emerging biomaterials due to their precise molecular design, biocompatibility, and ability to mimic natural protein functions. Polypeptides can be tailored to include bioactive motifs (e.g., RGD for cell adhesion) and self-assembling properties, forming hydrogels or nanofibers under physiological conditions (Li et al., 2023). Their tunable degradation—via enzymatic cleavage by proteases—and mechanical properties, combined with low immunogenicity, make them highly suitable for bio-ink applications, particularly in skin regeneration where cell signaling and ECM mimicry are critical. For example, a 3D-printed polypeptide-gelatin bio-ink with RGD motifs enhanced fibroblast proliferation and promoted wound closure in rat models (Masri and Fauzi, 2021). Another study utilized a self-assembling polypeptide bio-ink loaded with VEGF, which enhanced angiogenesis and promoted wound closure in porcine burn models.

Due to their programmable biochemical signalling capacity, polypeptide-based bio-inks may be particularly advantageous during proliferative phases requiring controlled cell recruitment, angiogenesis, and ECM organization.

### 3.3 Composite bio-ink materials

Composite bio-inks refer to a class of bio-inks fabricated by combining two or more distinct materials. This design strategy aims to integrate the strengths of individual components while mitigating their inherent limitations, thereby achieving enhanced overall performance. For instance, blending a naturally derived material with poor mechanical properties—such as collagen—with a printable, mechanically robust material like alginate can significantly improve printability and structural integrity. However, composite bio-inks do not simply combine material advantages; their design requires balancing biological activity, mechanical performance, printability, degradation behaviour, and phase-specific wound-healing requirements. Therefore, composite bio-inks represent a key strategy for achieving stage-matched functions that cannot be provided by single-component materials.

#### 3.3.1 Alginate-gelatin

Alginate-gelatin hydrogels are among the most widely utilized bio-inks in 3D skin bioprinting. This system integrates the rapid Ca^2+^-mediated gelation and favorable printability of alginate with the high biological activity of gelatin. Alginate serves as the primary structural framework, enabling immediate cross-linking via Ca^2+^ ions to form a stable hydrogel network that ensures precise shape fidelity during and after printing. In contrast, gelatin enhances cellular interactions by providing essential cell adhesion motifs and a supportive microenvironment for cell proliferation, thereby overcoming the inherent bio-inertness of pure alginate. By fine-tuning the alginate-to-gelatin ratio, an optimal balance between printability and biological functionality can be achieved, tailored to specific regenerative requirements (Monavari et al., 2023). Jia’s team employed 3D bioprinting technology to incorporate salvianolic acid B (SAB) into an alginate-gelatin matrix, fabricating a novel SAB-SA-Gel composite porous scaffold that exhibits potent antioxidant, anti-inflammatory, and pro-angiogenic properties in both cellular and rat models (Lihao et al., 2024).

These characteristics make alginate-gelatin composite bio-inks particularly suitable for the transition from the inflammatory to proliferative phase, where simultaneous regulation of oxidative stress, immune responses, and early tissue formation is required. Despite these advantages, the mechanical strength remains insufficient for large-scale wound applications, and degradation kinetics require further refinement.

#### 3.3.2 Collagen-alginate

The collagen-alginate composite system is designed to synergistically integrate the superior biological activity of collagen with the favorable mechanical properties and printability of alginate. Alginate effectively addresses the inherent limitations of pure collagen hydrogels—namely low mechanical strength and poor structural fidelity during printing—by forming a Ca^2+^-mediated crosslinking network that provides additional structural integrity. Conversely, the incorporation of collagen enriches the composite with abundant cell-adhesive motifs, such as RGD sequences, which counteract the bioinert nature of alginate and promote cell attachment, spreading, and functional activity (Hu and Lo, 2021).

The collagen-alginate bio-ink has been extensively investigated in skin regeneration. For example, collagen–alginate bio-inks have been used to fabricate bilayered skin models containing keratinocytes and fibroblasts, demonstrating good printability, structural organization, and support for cell viability and proliferation (Cavallo et al., 2023).

Given collagen’s role in ECM organization, collagen–alginate systems may also provide opportunities for regulating fibroblast behaviour and collagen remodelling during later stages of wound maturation. However, controlling excessive matrix deposition remains necessary to prevent fibrotic outcomes.

#### 3.3.3 GelMA-PEGDA

The GelMA-PEGDA composite hydrogel is a representative example of a natural–synthetic hybrid bio-ink, effectively combining the inherent biological activity of GelMA—derived from its collagen-like structure and cell-adhesive motifs—with the customizable mechanical properties of PEGDA, which can be precisely modulated through photopolymerization. This synergistic combination enables the fabrication of 3D-printed constructs with both high biocompatibility and robust mechanical resilience, making it highly suitable for a wide range of tissue engineering applications (Fowler et al., 2025). Furthermore, such composite hydrogels can provide controlled therapeutic delivery through tunable network properties (Chen Z. et al., 2023).

Collectively, composite bio-inks represent a compromise between biological functionality and engineering performance. Natural–natural combinations, such as alginate-gelatin and collagen-alginate systems, primarily enhance cell interaction and ECM-like signalling while improving printability. In contrast, natural–synthetic hybrids such as GelMA-PEGDA provide greater control over mechanical properties, crosslinking behaviour, and therapeutic release. Therefore, the optimal composite bio-ink depends not only on material compatibility but also on the biological demands of each wound-healing stage.

The biological functions, wound-healing phase relevance, and regenerative applications of representative bio-inks are further summarized in Table 3, providing a phase-oriented framework for bio-ink selection.

**TABLE 3:** Phase-specific biological roles and regenerative applications of representative bio-inks in skin wound healing.

Bio-ink	Dominant biological function	Relevant wound-healing phase(s)	Main regenerative application	Current challenge	Refs
Chitosan	Antibacterial activity, haemostasis, immune regulation	Haemostasis/Inflammatory phase	Infected wound management and inflammation control	Limited mechanical performance	(Bakshi et al., 2020; Kou et al., 2021; Lazaridou et al., 2022; Tiburcio et al., 2021; Elshabrawy et al., 2025; Taneja et al., 2022)
Alginate	Fluid absorption, haemostasis, moisture maintenance	Haemostasis/Early inflammation	Exudative wounds and cell/growth factor delivery	Poor intrinsic cell adhesion	(Lee and Mooney, 2012; Abasalizadeh et al., 2020; Bahú et al., 2022)
PVA	Moisture regulation and epithelial support	Inflammatory/Proliferative phases	Wound dressings and epithelial regeneration	Limited bioactivity	(Taneja et al., 2022; Teodorescu et al., 2018; Sun et al., 2021)
Cyclodextrin	Controlled therapeutic delivery	Inflammatory/Proliferative phases	Drug and growth-factor delivery systems	Needs structural support materials	(Zhang and Ma, 2013; Xue et al., 2023; Crini et al., 2018)
dECM	Tissue-specific signalling, macrophage modulation, angiogenesis	Inflammatory/Proliferative phases	Scar reduction and functional regeneration	Standardization issues	(Lai et al., 2024; Xiao et al., 2023; Jiang et al., 2023; Fu et al., 2023)
Hyaluronic acid	Cell migration, angiogenesis regulation, ROS modulation	Inflammatory resolution/Proliferative phases	Vascularized tissue regeneration	Rapid degradation	(Mirsky et al., 2024; Abalymov et al., 2022; Xu et al., 2020; Litwiniuk et al., 2016; Zhu et al., 2020)
Gelatin/GelMA	Cell adhesion, fibroblast migration, angiogenesis, ECM deposition	Proliferative phase	Dermal regeneration and vascularized skin substitutes	Requires crosslinking optimization	(C Echave et al., 2017; Bello et al., 2020; Yin et al., 2023; Hao et al., 2023; Abalymov et al., 2022)
Collagen	ECM mimicry, fibroblast activation, matrix formation	Proliferative/Remodeling phase	Dermal reconstruction and ECM remodeling	Weak mechanical stability	(Fernandez-Colino et al., 2019; Chattopadhyay and Raines, 2014; Chen et al., 2023a; Lazaridou et al., 2022; Zhe et al., 2023)
Polypeptides	Cell signalling and self-assembly	Proliferative phase	Biomimetic regenerative systems	Scalability and cost	(Masri and Fauzi, 2021; Li et al., 2023)
PEG	Controlled network formation and delivery	Proliferative/Remodeling phases	Smart hydrogel platforms	Bioinert nature	(Xie et al., 2025; Zhang et al., 2024; Ribeiro et al., 2024)
Silk fibroin	Mechanical reinforcement, structural support	Remodeling phase	Long-term tissue maturation and durable scaffolds	Limited biological signalling	(Kim et al., 2021; Agostinacchio et al., 2024; Amirian et al., 2024; Lai et al., 2024; Wu et al., 2025; Sultan et al., 2022)
PLA	Mechanical support, structural durability	Remodeling phase	Long-term scaffold formation	Poor biological interaction	(Farah et al., 2016; Taghizadeh et al., 2022; Lasprilla et al., 2012)
PLGA	Controlled degradation and factor delivery	Proliferative/Remodeling phases	Regenerative scaffolds and drug delivery	Acidic degradation effects	(Huon et al., 2020; Wu et al., 2021; lin et al., 2024)
PCL	Mechanical stability and long-term support	Remodeling phase	Durable skin substitutes	Slow degradation	(Woodruff and Hutmacher, 2010; Malikmammadov et al., 2018; Mokhena et al., 2024; Pugliese et al., 2021; Abdollahiyan et al., 2020; Radmanesh et al., 2022)
Cellulose	Structural reinforcement, hydration regulation	Remodeling phase	Mechanical stabilization of skin constructs	Low bioactivity	(Tiburcio et al., 2021; Eichhorn, 2011; Afewerki et al., 2019; Zheng et al., 2022)
Alginate-Gelatin	Antioxidant activity, angiogenesis, improved printability	Inflammatory → Proliferative transition	Chronic wound repair	Need for further mechanical optimization	(Hao et al., 2023; Lihao et al., 2024)
Collagen-Alginate	ECM mimicry with improved mechanics	Proliferative/Remodeling phases	Bilayered skin models	Long-term stability	(Hu and Lo, 2021; Cavallo et al., 2023)
GelMA-PEGDA	Tunable mechanics and controlled release	Proliferative/Remodeling phases	Vascularized tissue engineering	Fabrication complexity	(Fowler et al., 2025; Chen et al., 2023b)

### 3.4 Phase-specific selection of bio-inks for skin wound healing

Although no single bio-ink currently possesses all the characteristics required across the entire wound-healing process, rational selection should move beyond material classification and consider temporal compatibility between ink properties and the biological demands of each healing phase (Figure 2) (Zhang et al., 2022). The biological hallmarks of each phase have been detailed in Section 2 and Table 1; the following synthesis directly maps material categories to these phases based on the comprehensive descriptions in Sections 3.1–3.3.

**FIGURE 2:**
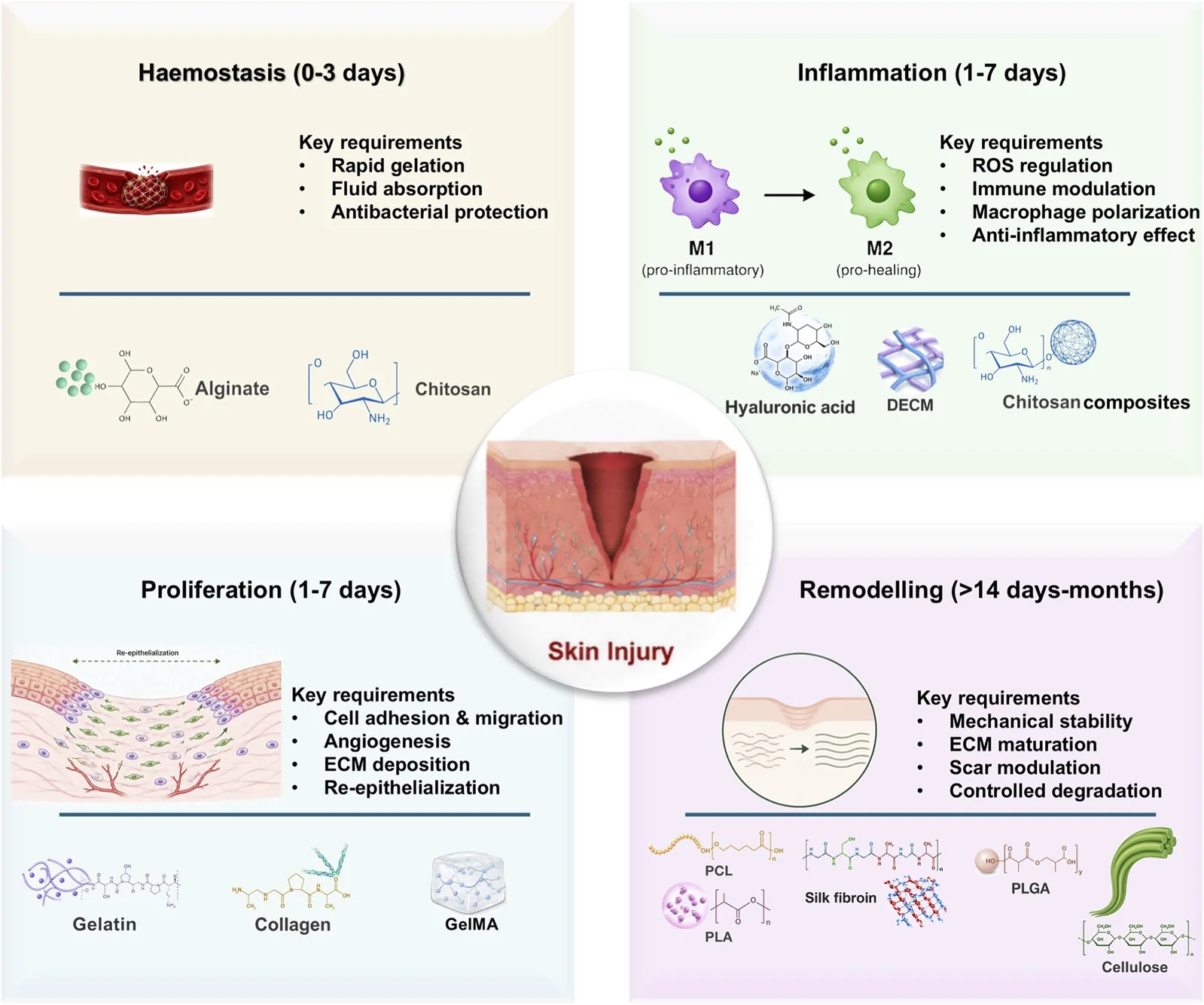
Phase-specific selection of bio-inks for skin wound healing.

Based on these properties, a phase-adaptive selection strategy can be distilled as follows: hemostasis and early inflammation favor alginate (instant ionic gelation, fluid absorption, exudate management) and chitosan (cationic antimicrobial action, hemostatic activity, and early immunomodulation), though their limited mechanics require composite reinforcement (Shi et al., 2026; Lee and Mooney, 2012; Yu et al., 2023); inflammatory resolution and transition are best supported by hyaluronic acid (ROS scavenging, CD44-mediated migration) and dECM (tissue-specific signals promoting M2 macrophage polarization), which regulate oxidative stress and temper excessive immune activation (Cui et al., 2026); the proliferative phase requires robust cell-material interactions, making collagen, GelMA, and HA ideal by providing RGD motifs and ECM-mimetic cues that drive fibroblast migration, angiogenesis, and keratinocyte proliferation, with GelMA offering additional tunability via photocrosslinking (Raihan et al., 2026; Das et al., 2024; Pazhouhnia et al., 2024); the remodelling phase demands long-term mechanical integrity and controlled matrix turnover, where PCL, PLA, PLGA, alongside silk fibroin and cellulose, provide durable support, though their limited bioactivity necessitates hybridization with ECM-derived components (Tian et al., 2025; Liu and Echeverry-Rendón, 2025). Since no single category satisfies all criteria, composite systems integrating biological signals with mechanical reinforcement represent the most practical current strategy.

Bio-ink development is shifting from material-centric design toward biologically guided, phase-adaptive strategies. Composite and smart-responsive platforms that integrate structural support, immune regulation, and spatiotemporal therapeutic delivery are promising, while future advances in 4D bioprinting, multi-omics guidance, and personalized manufacturing may enable inks that undergo programmed, time-dependent changes in mechanics and degradation to dynamically match the evolving repair microenvironment (Li et al., 2016; Derakhshanfar et al., 2018). Importantly, future 4D bio-inks are expected to evolve beyond passive responsiveness toward programmed, time-dependent changes in mechanical properties, degradation behaviour, and therapeutic release that correspond to specific stages of tissue repair.

## 4 Technological advances

The material properties of bio-inks dictate the feasible printing techniques, while technological innovations further broaden their application window. Importantly, the choice of printing technology is primarily governed by bio-ink rheology, target resolution, and scaffold geometry, rather than by wound-healing phases *per se*. Phase-specific biological functions are encoded in the bio-ink composition (see Section 3; Table 3), whereas printing techniques determine the feasibility of translating those compositions into structurally defined constructs. The following subsections focus on the technical capabilities, advantages, and limitations of each approach, without reiterating phase assignments already covered in Section 3.4.

### 4.1 Inkjet bioprinting

Inkjet bioprinting, a drop-on-demand printing technology, utilizes thermal or piezoelectric actuators to eject precise microdroplets of bio-ink onto a substrate, enabling high-resolution fabrication of tissue constructs (Li et al., 2016). Operating at droplet volumes as low as 1–100 pL and printing speeds up to 10,000 droplets per second, this method offers unparalleled precision (resolution <50 μm), making it ideal for creating intricate skin architectures with layered cellular patterns (Derakhshanfar et al., 2018). Its non-contact nature minimizes shear stress on cells, achieving viabilities above 90%, while its compatibility with low-viscosity bio-inks (1–20 mPa·s) facilitates the incorporation of natural and synthetic polymers. However, limitations such as nozzle clogging and restricted use with high-viscosity materials necessitate ongoing advancements, positioning inkjet bioprinting as a key technique for rapid prototyping and personalized wound repair applications.

Despite its excellent spatial resolution and high cell viability, the clinical translation of inkjet bioprinting remains constrained by its narrow printable viscosity range, which limits the use of mechanically robust or extracellular matrix-rich bio-inks. In addition, maintaining droplet stability while preserving homogeneous cell distribution becomes increasingly challenging when incorporating high cell densities or multifunctional biomaterials (Li et al., 2020). Consequently, recent research has shifted toward rheology-engineered bio-inks and hybrid bioprinting strategies that combine the precision of inkjet deposition with the structural advantages of extrusion-based systems, thereby expanding its applicability in complex skin tissue engineering.

Overall, inkjet bioprinting is particularly advantageous for applications requiring precise spatial cell patterning and rapid fabrication of superficial skin constructs. Nevertheless, its limited compatibility with high-viscosity bio-inks and mechanically demanding scaffolds continues to restrict its broader application in full-thickness skin engineering, highlighting the need for hybrid manufacturing strategies.

### 4.2 Extrusion-based bioprinting

Extrusion-based bioprinting, a widely adopted additive manufacturing technique, employs pneumatic or mechanical pressure to dispense continuous filaments of bio-ink through a nozzle, enabling the fabrication of complex 3D tissue constructs with high structural integrity. Operating with bio-inks of higher viscosity (30–6 × 10^7^ mPa·s) compared to inkjet methods, this approach supports a broad range of materials, including hydrogels and cell-laden composites, achieving resolutions of 100–500 μm and cell viabilities typically above 85% (Panwar and Tan, 2016). Its versatility in printing multi-material scaffolds and ability to incorporate high cell densities make it ideal for replicating the multilayered architecture of skin, though challenges such as shear stress on cells and slower printing speeds require optimization (Sun et al., 2020). Extrusion bioprinting’s scalability and compatibility with clinical-grade materials position it as a leading technology for large-scale wound repair and skin regeneration applications.

Despite its broad material compatibility and ability to fabricate mechanically stable constructs, extrusion bioprinting remains challenged by the shear stress generated during nozzle extrusion, which can adversely affect the viability and function of sensitive cell populations. In addition, the relatively low printing resolution compared with droplet-based or laser-assisted techniques limits the precise spatial organization of multiple skin cell types. Consequently, recent developments have focused on optimizing nozzle geometry, extrusion parameters, and shear-thinning bio-inks to balance print fidelity, mechanical stability, and cellular functionality in engineered skin substitutes. Across the landscape of wound healing, extrusion-based bioprinting reveals remarkable promise. For instance, a study utilized an extrusion-printed alginate-gelatin bio-ink loaded with VEGF, demonstrating enhanced angiogenesis and wound closure through sustained VEGF delivery in porcine burn models within 21 days, attributed to its ability to create vascularized scaffolds. Another investigation employed a collagen-chitosan bio-ink via extrusion bioprinting, reducing wound area by 40% in rat models within 14 days and enhancing collagen deposition by 25%, owing to its precise layering and structural support (Naghieh and Chen, 2021).

Taken together, these findings demonstrate that extrusion-based bioprinting remains the most versatile platform for fabricating clinically relevant skin substitutes because of its compatibility with highly viscous and cell-laden bio-inks. Nevertheless, further optimization is required to minimize shear-induced cell damage while improving printing resolution and vascular complexity.

### 4.3 Laser-assisted bioprinting

Laser-assisted bioprinting (LAB), a nozzle-free bioprinting technique, employs a laser pulse to transfer bio-ink droplets from a donor ribbon to a substrate, offering high precision and cell viability for tissue engineering applications (Guillotin et al., 2013). Utilizing a laser-induced forward transfer mechanism, LAB achieves resolutions as fine as 10–20 μm and printing speeds of 200–1,600 mm/s, making it ideal for fabricating delicate skin constructs with intricate cellular arrangements (Yang et al., 2021).

Its ability to handle a wide range of bio-ink viscosities (1–300 mPa·s) and maintain cell viabilities above 95%—due to minimal mechanical stress—distinguishes it from other methods, though its high cost and complexity limit scalability. LAB’s precision and compatibility with cell-laden bio-inks position it as a cutting-edge technology for personalized skin regeneration and wound repair. Although LAB provides exceptional printing precision and minimizes mechanical damage to encapsulated cells, its widespread implementation is hindered by high equipment costs, limited throughput, and the technical complexity of laser calibration. These challenges currently restrict its application primarily to experimental and small-scale tissue fabrication. Therefore, improving system scalability while maintaining printing accuracy remains a major objective for future clinical translation.

Amidst the domain of wound restoration, LAB unveils striking effectiveness. For instance, a study used LAB to print a collagen-hyaluronic acid bio-ink, achieving a 30% increase in fibroblast viability within 5 days and reducing wound area by 35% in rat models within 14 days, attributed to its accurate cell patterning and ECM mimicry. Another investigation employed LAB with a gelatin-based bio-ink loaded with keratinocytes, enhancing re-epithelialization by 40% and achieving 85% wound closure in porcine models within 21 days, highlighting its capacity to create stratified skin layers (Guillotin et al., 2013). Such evidence reveals LAB’s prowess in generating refined skin substitutes, propelling sophisticated repair paradigms.

### 4.4 Stereolithography

Stereolithography (SLA), a light-based bioprinting technique, utilizes photopolymerization to selectively cure liquid bio-inks layer-by-layer, offering unmatched resolution and speed for tissue engineering applications (Kumar and Kim, 2020). Employing UV or visible light to crosslink photosensitive resins—typically containing acrylate or methacrylate groups—SLA achieves resolutions below 10 μm and printing times reduced by up to 100-fold compared to extrusion methods, making it ideal for fabricating complex skin structures (Zhu et al., 2017). Its high precision supports the creation of microscale features, such as vascular channels, while maintaining cell viability above 90%, though it is limited to photocrosslinkable bio-inks and requires careful optimization to avoid cytotoxicity from photoinitiators (Lim et al., 2018). SLA’s ability to produce smooth, mechanically robust scaffolds positions it as a transformative technology for rapid skin regeneration and wound repair. Despite its exceptional printing resolution and geometric accuracy, the clinical application of SLA remains dependent on the availability of biocompatible photocrosslinkable bio-inks and photoinitiators with minimal cytotoxicity. Furthermore, balancing rapid photocuring with long-term cellular function continues to represent a critical challenge in skin tissue engineering. Recent advances in visible-light photopolymerization and naturally derived photoresponsive biomaterials are expected to improve the biological performance and translational potential of SLA-based skin constructs.

SLA has emerged as a promising technique with substantial impact in real-world wound healing scenarios. For instance, a study used SLA to print a gelatin-methacryloyl (GelMA) bio-ink, achieving a 25% increase in fibroblast proliferation within 5 days and reducing wound area by 30% in rat models within 14 days, attributed to its precise scaffold architecture (Pazhouhnia et al., 2024). Another investigation employed SLA with a hyaluronic acid-methacrylate (HAMA) bio-ink loaded with VEGF, enhancing angiogenesis by 35% and achieving 85% wound closure in porcine models within 21 days, highlighting its capacity to support vascularized skin regeneration (Kumar and Kim, 2020).

These instances highlight the effectiveness of SLA in generating high-quality skin substitutes suitable for advanced tissue repair. Collectively, current evidence indicates that stereolithography is particularly suitable for fabricating highly organized skin constructs with microscale architectural precision. Nevertheless, continued development of safer photopolymer systems and bioactive photocurable bio-inks will be essential for its broader clinical translation.

### 4.5 Future directions: smart, adaptive, and data-driven bio-inks

Emerging trends in bioprinting are revolutionizing skin tissue engineering by integrating advanced materials, multi-omics data, and hybrid technologies to address the dynamic complexity of wound repair (Li et al., 2016; Sun et al., 2020). Innovations such as four-dimensional (4D) bioprinting have introduced the concept of temporally adaptive constructs, in which printed architectures are designed to undergo controlled changes over time in response to biological or environmental cues. Although many current 4D bio-inks rely on passive stimulus-responsive behaviours, future developments are expected to move toward programmed, time-dependent regulation of mechanical properties, degradation profiles, and therapeutic release to better match the sequential requirements of wound healing (Sun et al., 2020). Additionally, the incorporation of smart bio-inks with embedded sensors or stimuli-responsive nanoparticles enhances real-time monitoring and controlled release of therapeutics, while machine learning-guided bioprinting optimizes scaffold design for patient-specific regeneration (Fan et al., 2024). These advancements, combined with stem cell-laden bio-inks and vascularized constructs, may help address current limitations in scalability, functionality, and clinical translation, further advancing the role of bioprinting in regenerative skin medicine.

One promising Frontier is the integration of multi-omics datasets into 4D bioprinting workflows. Recent single-cell transcriptomic studies of human skin wound repair have revealed dynamic, stage-specific shifts—for example, early upregulation of pro-inflammatory cytokines such as TNF-α and IL-6 during the inflammatory phase, followed by increased expression of angiogenic and ECM-remodeling genes including VEGFA and COL1A1 during proliferative and remodeling phases (Liu et al., 2025). Incorporating these temporal molecular signatures into bio-ink design enables stimuli-responsive formulations that release therapeutic cues in synchrony with each healing stage, thereby supporting a transition toward more data-driven and adaptive bio-ink design strategies.

These emerging strategies have demonstrated the potential of adaptive bio-inks to dynamically regulate cellular organization, vascular development, and tissue regeneration. For example, moisture-responsive 4D bioprinted GelMA-based systems have shown improved fibroblast organization and accelerated wound closure in preclinical models by adapting to changes in the wound microenvironment (Yin et al., 2023; Jiang et al., 2025). Similarly, computationally guided smart PLGA-based bio-inks incorporating VEGF-releasing nanoparticles have enhanced vascular formation and promoted tissue regeneration through controlled therapeutic delivery (Costa et al., 2021). Collectively, these findings highlight the potential of advanced bioprinting strategies to improve the functional complexity and translational potential of engineered skin substitutes. Various bioprinting techniques, including Inkjet, Extrusion, Laser-assisted Bioprinting (LAB), and Stereolithography (SLA), differ in their resolution capabilities, while emerging 4D bioprinting introduces dynamic adaptability (Figure 3). A detailed comparison of bioprinting techniques, including their key properties, advantages, and limitations, is provided in Table 4.

**FIGURE 3:**
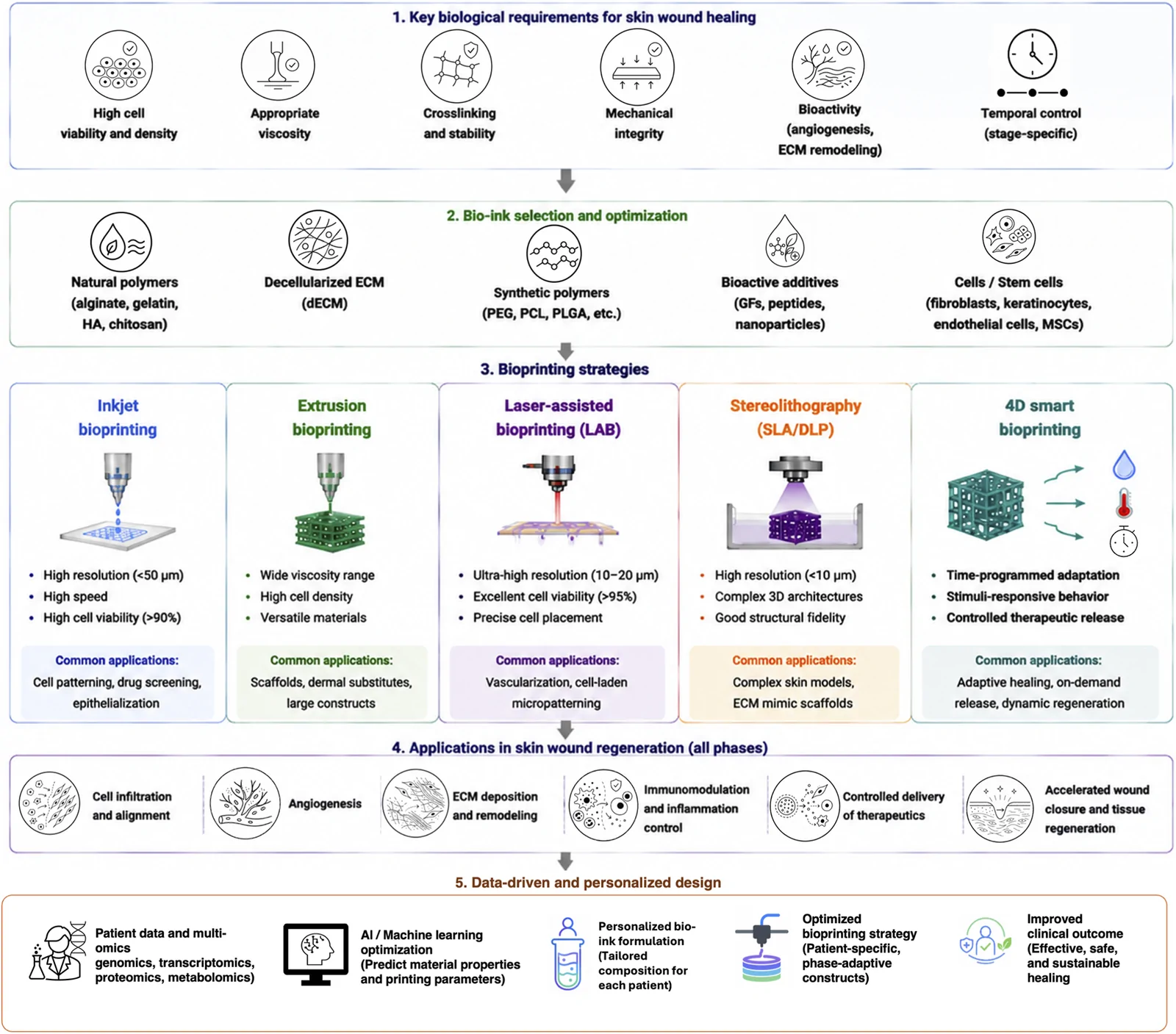
Comparison of bioprinting techniques for skin tissue engineering. Resolution comparison of established techniques (Inkjet, Extrusion, LAB, SLA) alongside the emerging trend of 4D bioprinting, where scaffolds adapt to environmental stimuli (e.g., moisture, shown by a droplet) over time (indicated by a clock), enhancing cell alignment.

**TABLE 4:** Comparison of bioprinting technologies for skin tissue engineering.

Technique	Source/Type	Key properties	Advantages	Limitations	Refs
Inkjet bioprinting	Droplet-based	High resolution (<50 μm), >90% viability	High speed cost-effective	Limited viscosity range and thermal stress	(Li et al., 2016; Alves et al., 2021)
Extrusion-based bioprinting	Filament-based	High viscosity (30–6 × 10^7^ mPa s), >85% viability	High cell density, versatility	Shear stress, lower resolution	(Xie et al., 2021)
Laser-assisted bioprinting	Laser-induced transfer	Ultra-high resolution (10–20 μm), >95% viability	High precision and excellent cell viability	High cost Limited scalability	(Guillotin et al., 2013; Deng et al., 2022)
Stereolithography	Photopolymerization	Sub-10 μm resolution, >90% viability	High accuracy, complex scaffolds	Cytotoxicity Limited compatibility with photocrosslinkable bio-inks	(Pazhouhnia et al., 2024; Grigoryan et al., 2019)
Emerging trends (e.g., 4D)	Temporally adaptive/stimuli-responsive	Time-dependent structural and functional adaptation	Dynamic response, personalized and stage-specific therapeutic regulation	Limited bio-inks availability, complex fabrication and validation requirements	(C Echave et al., 2017; Sun et al., 2020)

Ultimately, future bioprinting strategies are expected to integrate material intelligence, temporal biological information, and advanced manufacturing technologies to generate phase-adaptive skin substitutes capable of dynamically supporting the entire wound-healing cascade.

## 5 Multifunctionality of bio-ink in wound repair

In the context of the phase-specific framework, these functions are not independent but strategically timed. Antibacterial and anti-inflammatory actions are paramount during the hemostasis and early inflammation phases to prevent infection and excessive immune activation. Pro-angiogenic activity becomes critical during the proliferative phase to support granulation tissue formation. Finally, fibrosis modulation is a key goal during the remodeling phase to prevent pathological scarring (Table 5). The detailed material compositions, formulation strategies, and preclinical evidence for each function have been comprehensively presented in Sections 3.1–3.3 and are summarized in Tables 2, 3. The present section focuses solely on the mechanistic principles, stage-specific timing, and shared design challenges of these functions, without reiterating individual material case studies already covered above.

**TABLE 5:** Functional roles of bio-inks across wound healing phases.

Function	Main phase	Key mechanisms	Representative bio-inks (see Sections 3.1–3.3 for details)
Antibacterial	Haemostasis/inflammation	microbial control	Chitosan, Ag-based systems
Anti-inflammatory	Inflammation	ROS regulation, macrophage modulation	HA, dECM
Angiogenesis	Proliferation	VEGF delivery, vascular formation	GelMA, alginate composites
Scar modulation	Remodeling	TGF-β regulation, ECM balance	HA, collagen composites

### 5.1 Antibacterial function

The antibacterial functionality of bio-inks is critical in wound repair, as microbial contamination remains a primary cause of delayed healing and chronic infections, particularly in burns and diabetic ulcers (Develioglu et al., 2020). The antibacterial function is primarily achieved through cationic polymers (e.g., chitosan disrupting bacterial membranes), metallic nanoparticles (e.g., silver ions), or antimicrobial peptides that inhibit biofilm formation and reduce pathogen load without relying solely on systemic antibiotics (Tiburcio et al., 2021). This localized action is particularly valuable for burn and diabetic ulcer management (Rybka et al., 2022; Rastin et al., 2021). However, excessive concentrations of metallic or cationic agents may induce cytotoxicity toward mammalian cells and interfere with tissue remodeling. Therefore, current research increasingly emphasizes controlled-release systems and non-antibiotic antimicrobial strategies to balance efficacy with long-term biocompatibility. (Specific formulation examples and preclinical data are detailed in Sections 3.1.2 for chitosan and 3.2 for synthetic composite systems.)

### 5.2 Anti-inflammatory and antioxidant functions

Anti-inflammatory and antioxidant functionalities mitigate excessive inflammation and oxidative stress driven by reactive oxygen species (ROS), which prolong healing and exacerbate tissue damage in chronic wounds. Bio-inks achieve this by incorporating ROS-scavenging materials (e.g., hyaluronic acid), anti-inflammatory agents (e.g., curcumin), or polymers that promote macrophage polarization from pro-inflammatory (M1) to pro-healing (M2) phenotypes, thereby reducing TNF-α and IL-6 levels while neutralizing free radicals (Zhu et al., 2020; Barchitta et al., 2019; Ding et al., 2023). The precise spatial control offered by 3D bioprinting further enhances efficacy by targeting these therapeutic components to specific wound regions, making such bio-inks essential for managing inflammation-driven pathologies in skin regeneration. Importantly, excessive suppression of ROS may impair normal cell signaling required during the early inflammatory phase (Datta et al., 2019). Therefore, contemporary bio-ink design aims to regulate rather than completely eliminate oxidative stress, thereby maintaining physiological redox signaling while preventing chronic inflammation. (The specific mechanisms of HA and dECM are covered in Sections 3.1.2 and 3.1.3, respectively.).

### 5.3 Angiogenesis promotion

Angiogenesis promotion is essential for delivering oxygen and nutrients to regenerating skin, particularly in large or chronic wounds. Bio-inks enhance vascularization by incorporating pro-angiogenic factors (e.g., VEGF), co-culturing endothelial cells, or designing microchannel-forming materials that stimulate endothelial migration, proliferation, and tubule formation within printed scaffolds (Xie et al., 2021; Yeo et al., 2023). A major challenge is that uncontrolled VEGF delivery often results in immature and poorly organized vascular networks; hence, recent strategies emphasize spatiotemporal growth factor release, endothelial cell co-culture, or microvascular patterning to achieve stable and functional neovasculature (Pajooh et al., 2024; Liao et al., 2023; Li et al., 2024). (Representative pro-angiogenic composite systems, such as VEGF-loaded alginate-gelatin, are described in Section 3.3.1.)

### 5.4 Fibrosis modulation

Fibrosis modulation aims to prevent excessive scar formation driven by overactive myofibroblast differentiation and pathological collagen deposition, which compromise skin function and aesthetics. Bio-inks achieve this by incorporating anti-fibrotic agents (e.g., TGF-β modulators), modulating ECM homeostasis, or promoting balanced macrophage activity that favors regeneration over scarring (Guo and Longaker, 2023; Zhang et al., 2025). This approach reduces fibrogenic markers (α-SMA, collagen I) and mitigates mechanical stiffness (Briones et al., 2025). Importantly, complete suppression of fibrotic activity is undesirable because controlled ECM deposition is essential for restoring tissue integrity; therefore, emerging strategies focus on modulating fibroblast activation and ECM remodeling rather than abolishing fibrosis entirely. (Specific anti-fibrotic examples, including TGF-β inhibitor-loaded gelatin and curcumin-loaded cyclodextrin systems, have been detailed in Sections 3.1.1 and 3.1.2.)

## 6 Clinical applications and case studies

The translation of bio-ink technologies into clinical applications represents a transformative Frontier in wound repair, bridging laboratory innovations with patient-specific therapeutic outcomes (Murphy and Atala, 2014). Bioprinted scaffolds, leveraging the multifunctional properties of bio-inks—antibacterial action, angiogenesis promotion, inflammation modulation, and fibrosis modulation—offer tailored solutions for complex wounds, such as burns, diabetic ulcers, and surgical defects, where conventional treatments often fall short (Li et al., 2016). Despite these advances, successful clinical translation depends not only on therapeutic efficacy but also on reproducible bio-ink manufacturing, sterilization strategies, standardized printing protocols, regulatory approval, commercialization feasibility, and long-term safety evaluation. Addressing these challenges is essential for the widespread clinical adoption of bioprinted skin substitutes.

### 6.1 Current clinical progress and translational barriers

Recent clinical-oriented research illustrates tangible progress. In a patient-oriented study, 3D-bioprinted autologous MA-ECM constructs for diabetic foot ulcers demonstrated improved wound closure outcomes compared with standard care, supporting the feasibility of personalized bioprinting for chronic wound management (Kesavan et al., 2024). Parallel to this, a programmable handheld extrusion-based bioprinting platform has been introduced, enabling *in situ* wound dressing under smartphone control and offering flexible deployment in emergency or bedside settings (Wang et al., 2024). Additionally, 3D-printed bio-inks applied to diabetic ulcer contexts have shown enhanced cell activity, angiogenesis, and scaffold integrity, pointing toward tangible clinical benefits (Abuhamad et al., 2024).

Collectively, these studies reveal that clinical translation is progressing through two complementary strategies: (1) patient-specific fabrication for personalized wound treatment, and (2) multifunctional bio-ink systems designed to regulate biological processes such as vascularization and tissue remodeling. Importantly, successful translation will likely depend on integrating these approaches with phase-specific bio-ink design principles rather than pursuing a universal bio-ink platform.

Despite encouraging results, current evidence remains preliminary. Most studies involve small patient cohorts, lack randomized controlled designs, and are limited by short follow-up periods (typically ≤12 months), restricting evaluation of long-term outcomes such as scar maturation and functional skin regeneration. Furthermore, variability in wound type, patient comorbidities, and printing protocols introduces heterogeneity, while regulatory and manufacturing challenges continue to slow standardized adoption (Kumar and Kim, 2020).

Beyond clinical validation, large-scale production of bio-inks remains challenging due to batch-to-batch variability of biological materials, sterilization requirements, storage stability, and the need for reproducible printing performance. Furthermore, regulatory pathways for cell-laden and multifunctional bio-inks remain complex, as these products often combine characteristics of biomaterials, medical devices, and advanced therapeutic products. Addressing these translational barriers will be essential for moving bio-ink technologies from experimental platforms toward routine clinical applications.

### 6.2 Preclinical and early clinical evidence

Several clinical investigations and preclinical studies have demonstrated the potential of bio-ink-based interventions across different wound types and bioprinting platforms. Extrusion-based bioprinting using alginate-based bio-inks has shown promise in fabricating hydrated, structurally supportive wound scaffolds, with favorable gelation characteristics and tunable properties that support their incorporation into composite systems (Rafieyan et al., 2024). Recent computational approaches have further improved bio-ink optimization by enabling prediction of scaffold quality and printing outcomes, supporting more reproducible fabrication (Rafieyan et al., 2024).

A graphene-reinforced nanocomposite bio-ink demonstrated enhanced granulation tissue formation and wound closure in diabetic wound models, suggesting potential for chronic wound management (Pahlevani et al., 2025; Bukit et al., 2024). Multifunctional antibacterial systems incorporating chitosan matrices and silver nanoparticles have shown promising results in infected wound models by combining antimicrobial activity with enhanced tissue regeneration, reducing bacterial burden while supporting granulation tissue formation and collagen remodeling.

*In situ* bioprinting using cell-laden bio-inks has also demonstrated significant potential. For example, an autologous skin cell-laden bio-ink applied directly to full-thickness wounds in a porcine model resulted in accelerated wound closure, enhanced vascularization, and improved dermal regeneration, highlighting the translational potential of personalized bioprinting approaches (Albanna et al., 2019).

Taken together, these clinical and preclinical studies highlight the therapeutic potential of bio-inks while emphasizing the need for standardized protocols, larger clinical studies, and long-term evaluation before routine clinical implementation. Bio-inks play a multifunctional role in wound repair, providing structural support, enhancing cell growth, and accelerating wound closure, among other benefits (Figure 4).

**FIGURE 4:**
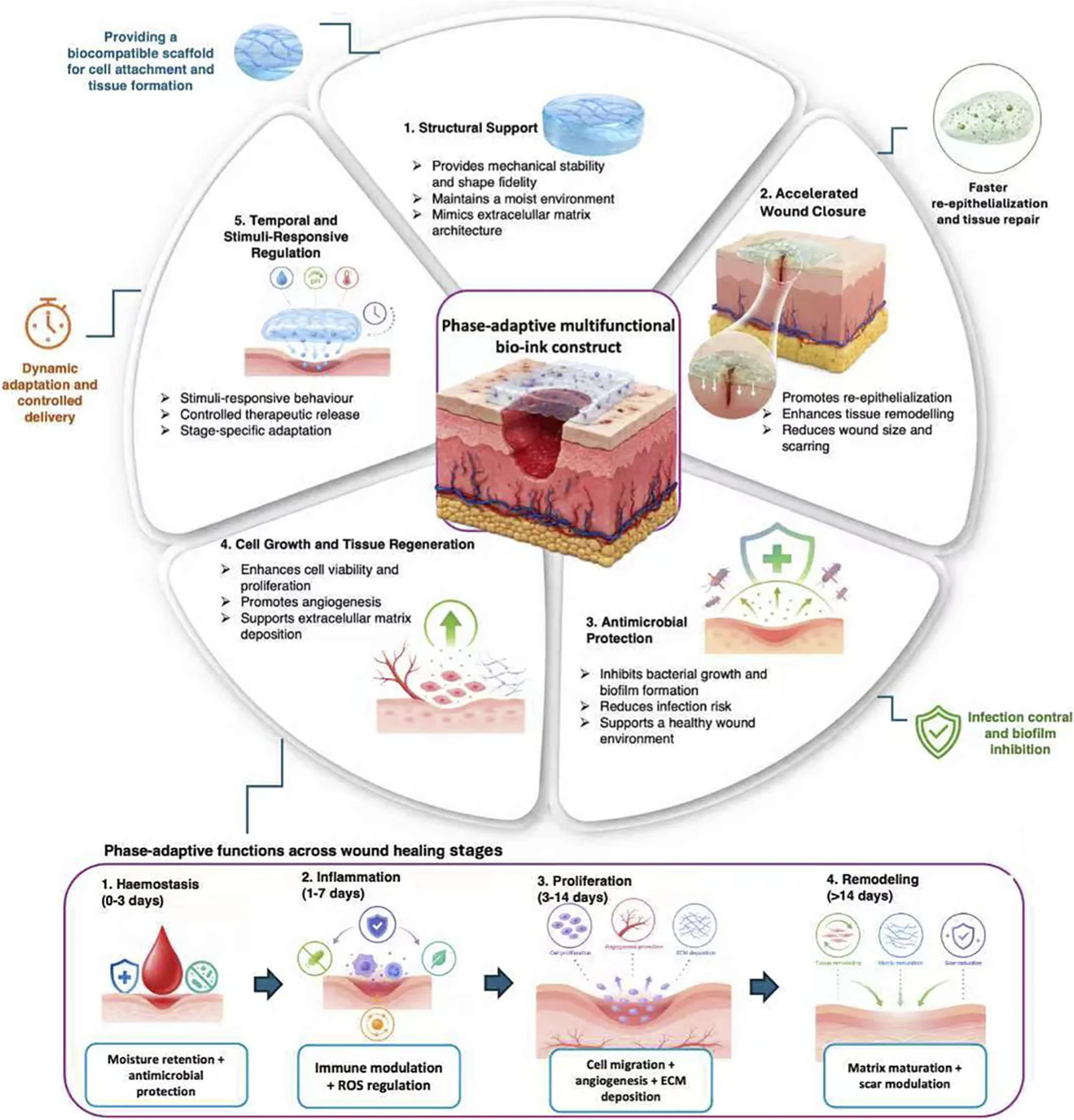
Multifunctional and phase-adaptive roles of bio-inks in skin wound repair. Bio-inks integrate structural support with biological regulation by enhancing cell growth, promoting wound closure, providing antimicrobial protection, and enabling adaptive responses to dynamic wound microenvironments.

## 7 Conclusion and future perspectives

Bio-ink technologies have revolutionized wound healing and skin regeneration by integrating antibacterial, pro-angiogenic, anti-inflammatory, and anti-fibrotic properties into patient-specific bioprinted constructs, representing a significant advancement beyond conventional wound management approaches. Current successes, such as enhanced vascularization and infection control, underscore their potential to accelerate healing and improve functional outcomes in burns, diabetic ulcers, and traumatic injuries. Nevertheless, persistent challenges—namely, the replication of intricate microvascular networks, skin appendages (e.g., hair follicles and sweat glands), and biomechanical equivalence to native tissue—limit full restoration. However, no single bio-ink currently satisfies all biological and engineering requirements across the entire wound-healing process, and optimal strategies are likely to involve phase-adaptive or composite systems rather than a universal material platform. Addressing these gaps requires a convergence of materials science, bioprinting innovation, and translational research, positioning bio-inks as a cornerstone of next-generation regenerative medicine.

Looking ahead, several emerging technologies hold potential to substantially enhance bio-ink functionality and clinical applicability. 4D bioprinting has enabled stimuli-responsive systems—such as GelMA-based bio-inks that adapt to pH and hydration changes—that better match the dynamic wound microenvironment (Wang M. et al., 2022). *In situ* bioprinting allows direct deposition of cell-laden bio-inks onto wound sites, with preclinical models demonstrating improved closure and regeneration, suggesting bedside applicability (Liu et al., 2024). Microfluidic-assisted strategies have advanced vascular network formation and microstructural control, addressing critical limitations in nutrient transport and tissue organization (Englert et al., 2025). Cell-based approaches, including fibroblast therapies, offer new avenues for regulating ECM deposition and scar modulation, though their integration with bio-ink platforms remains an important future direction (Rahnama et al., 2024). Perhaps most significantly, multi-omics-guided bioprinting integrates molecular-level data with biomaterial design to personalize regenerative therapies. For instance, transcriptomic profiling of growth factors (VEGFA, FGF2, TGF-β1) across healing stages can inform stimulus-responsive release modules; proteomic mapping of ECM proteins (collagen I/III ratio, laminins) can guide adjustments in stiffness and degradation kinetics; and metabolomic insights into oxidative stress can direct the incorporation of ROS-sensitive or oxygen-releasing components. Together, these approaches illustrate how 4D bio-inks, programmed by multi-omics datasets, can evolve into truly stage-adaptive systems (Saha et al., 2026).

Beyond these, the incorporation of artificial intelligence (AI) and nanotechnology offers transformative potential. AI-driven models have shown potential to optimize bio-ink formulations by balancing viscosity, cell viability, and degradation behavior, thereby accelerating biomaterial design workflows (Rafieyan et al., 2024). Meanwhile, nanoparticle-reinforced bio-inks have demonstrated improved mechanical properties and antibacterial performance, highlighting their potential for chronic wound applications (Bukit et al., 2024).

Despite significant advancements, several practical challenges hinder the transition of bio-ink technologies into standard clinical practice. First, regulatory approval (e.g., from the FDA or EMA) remains a complex and costly process due to the need for long-term biocompatibility assessments and standardized bioprinting protocols; for instance, advanced clinical evaluation may require extensive time and financial resources. Second, high production costs associated with bioactive materials, cell processing, and advanced printing systems is prohibitively high for widespread adoption, driven by the expense of high-purity raw materials (e.g., pharmaceutical-grade collagen or synthetic polymers) and advanced bioprinting equipment. Third, the stability of bio-inks under real-world conditions—such as long-term storage or transportation—remains underexplored; materials like gelatin or chitosan may degrade or lose viscosity at elevated temperatures, compromising printability and efficacy. Fourth, equitable access is limited by the concentration of bioprinting technology in developed regions and the lack of infrastructure in underserved areas, posing a socio-economic barrier. To address these, cost-effective bio-inks (e.g., based on abundant cellulose or alginate), scalable and affordable bioprinting platforms (e.g., open-source printers), and international collaborations for technology transfer are proposed as viable solutions. By addressing these through scalable bioprinting platforms and open-source bio-ink formulations, bio-ink technologies could transition from experimental platforms to standard clinical tools, facilitating the development of more functional and clinically applicable skin substitutes in the coming years. (Huang et al., 2024). From a material-selection perspective, current evidence suggests that ECM-derived bio-inks such as collagen, GelMA, and dECM provide superior biological signalling for cell integration and vascularized regeneration, whereas synthetic polymers such as PCL, PLA, and PLGA offer mechanical reinforcement and long-term structural stability. Composite bio-inks that combine biological activity with programmable mechanical properties are therefore likely to represent the most practical direction for future skin regeneration applications.

In conclusion, the field of bio-ink development for skin regeneration has progressed from a purely material-centric discipline to a more biologically informed endeavor. The future of this field lies not in the discovery of a single ‘ideal’ bio-ink, but in the intelligent design of adaptive, phase-specific systems. By integrating the principles of wound biology, advanced manufacturing, and data-driven design, the next-generation of bio-inks holds the potential to transform wound care from passive repair to active, temporally programmed regeneration, ultimately overcoming the persistent challenges of chronic wounds and pathological scarring.
